# Age, comorbidity, life expectancy, and pulmonary nodule follow-up in older veterans

**DOI:** 10.1371/journal.pone.0200496

**Published:** 2018-07-25

**Authors:** Melisa L. Wong, Ying Shi, Kathy Z. Fung, Sarah Ngo, Brett M. Elicker, James K. Brown, Robert A. Hiatt, Victoria L. Tang, Louise C. Walter

**Affiliations:** 1 Division of Hematology/Oncology, Helen Diller Family Comprehensive Cancer Center, University of California, San Francisco, San Francisco, CA, United States of America; 2 Division of Geriatrics, University of California, San Francisco and San Francisco Veterans Affairs Medical Center, San Francisco, CA, United States of America; 3 Department of Radiology and Biomedical Imaging, University of California, San Francisco and San Francisco Veterans Affairs Medical Center, San Francisco, CA, United States of America; 4 Pulmonary, Critical Care, and Sleep Medicine Section, San Francisco Veterans Affairs Medical Center, San Francisco, CA, United States of America; 5 Department of Epidemiology and Biostatistics, University of California, San Francisco, San Francisco, CA, United States of America; West Virginia University, UNITED STATES

## Abstract

**Background:**

Pulmonary nodule guidelines do not indicate how to individualize follow-up according to comorbidity or life expectancy.

**Objectives:**

To characterize comorbidity and life expectancy in older veterans with incidental, symptom-detected, or screen-detected nodules in 2008–09 compared to 2013–14. To determine the impact of these patient factors on four-year nodule follow-up among the 2008–09 subgroup.

**Design:**

Retrospective cohort study.

**Setting:**

Urban Veterans Affairs Medical Center.

**Participants:**

243 veterans age ≥65 with newly diagnosed pulmonary nodules in 2008–09 (followed for four years through 2012 or 2013) and 446 older veterans diagnosed in 2013–14.

**Measurements:**

The primary outcome was receipt of any follow-up nodule imaging and/or biopsy within four years after nodule diagnosis. Primary predictor variables included age, Charlson-Deyo Comorbidity Index (CCI), and life expectancy. Favorable life expectancy was defined as age 65–74 with CCI 0 while limited life expectancy was defined as age ≥85 with CCI ≥1 or age ≥65 with CCI ≥4. Interaction by nodule size was also examined.

**Results:**

From 2008–09 to 2013–14, the number of older veterans diagnosed with new pulmonary nodules almost doubled, including among those with severe comorbidity and limited life expectancy. Overall among the 2008–09 subgroup, receipt of nodule follow-up decreased with increasing comorbidity (CCI ≥4 versus 0: adjusted RR 0.61, 95% CI 0.39–0.95) with a trend towards decreased follow-up among those with limited life expectancy (adjusted RR 0.69, 95% CI 0.48–1.01). However, we detected an interaction effect with nodule size such that comorbidity and life expectancy were associated with decreased follow-up only among those with nodules ≤6 mm.

**Conclusions:**

We found some individualization of pulmonary nodule follow-up according to comorbidity and life expectancy in older veterans with smaller nodules only. As increased imaging detects nodules in sicker patients, guidelines need to be more explicit about how to best incorporate comorbidity and life expectancy to maximize benefits and minimize harms for patients with nodules of all sizes.

## Introduction

Over 1.5 million Americans are diagnosed with incidental pulmonary nodules annually [1]. With implementation of lung cancer screening and new indications for CT imaging, even more patients will be diagnosed with nodules, especially older adults. For example, the US Preventive Services Task Force (USPSTF) lung cancer screening recommendation [2] focuses on high-risk adults age 55 to 80 years. Neither the Fleischner Society guidelines [3, 4] for incidental nodules nor the Lung Computed Tomography (CT) Screening Reporting and Data System (Lung-RADS) [5] guideline for screen-detected nodules provide detailed information on how to incorporate age, comorbidity, or life expectancy to individualize nodule follow-up. As increasing numbers of older patients and their providers face these clinical challenges, it is important to characterize comorbidity and life expectancy in older adults with pulmonary nodules and how these patient factors impact nodule follow-up.

Prior studies on the impact of age and comorbidity on pulmonary nodule follow-up have been limited. A retrospective study of veterans with incidental or symptomatic nodules in 2003–06 found that older age was associated with less intensive follow-up than recommended by guidelines [6]. However, chronic obstructive pulmonary disease (COPD) was the only comorbidity examined. Another retrospective study of incidental pulmonary nodules diagnosed in 2007 found that follow-up nodule imaging did not differ by age [7]. However, this study did not examine any comorbidities. No studies have examined the impact of multiple comorbidities or life expectancy on nodule follow-up.

We performed a retrospective cohort study to a) characterize age, comorbidity, and life expectancy among veterans age ≥65 with newly diagnosed incidental, symptom-detected, or screen-detected pulmonary nodules in 2008–09 compared to 2013–14 and b) determine the impact of age, comorbidity, and life expectancy on four-year follow-up among the 2008–09 subgroup.

## Methods

### Data sources and patients

We identified veterans age ≥65 years with newly diagnosed pulmonary nodules (solitary or multiple) on CT chest scan at the San Francisco Veterans Affairs Medical Center (SFVAMC) in 2008–09 and 2013–14. The years 2008–09 were selected to represent patterns of pulmonary nodule diagnosis and evaluation prior to the 2013 USPSTF lung cancer screening recommendation [2]. The years 2013–14 were selected to represent patterns of pulmonary nodule diagnosis after the screening recommendation changed. At the SFVAMC, patients with high-risk nodules are referred to chest clinic while those with low-risk nodules are managed by primary care with assistance from a nurse practitioner-led nodule tracking program.

We queried radiology CT chest reports with search terms “nodul*” and/or “mass” and manually reviewed to confirm the presence of a new pulmonary nodule. Exclusion criteria included patients with a history of invasive cancer, pulmonary nodules previously identified on CT imaging, nodules of other organs (e.g. thyroid), and nodules with benign patterns of calcification (e.g., granulomas). Demographics and vital status were obtained from the VA National Patient Care Database.

**Fig 1:**
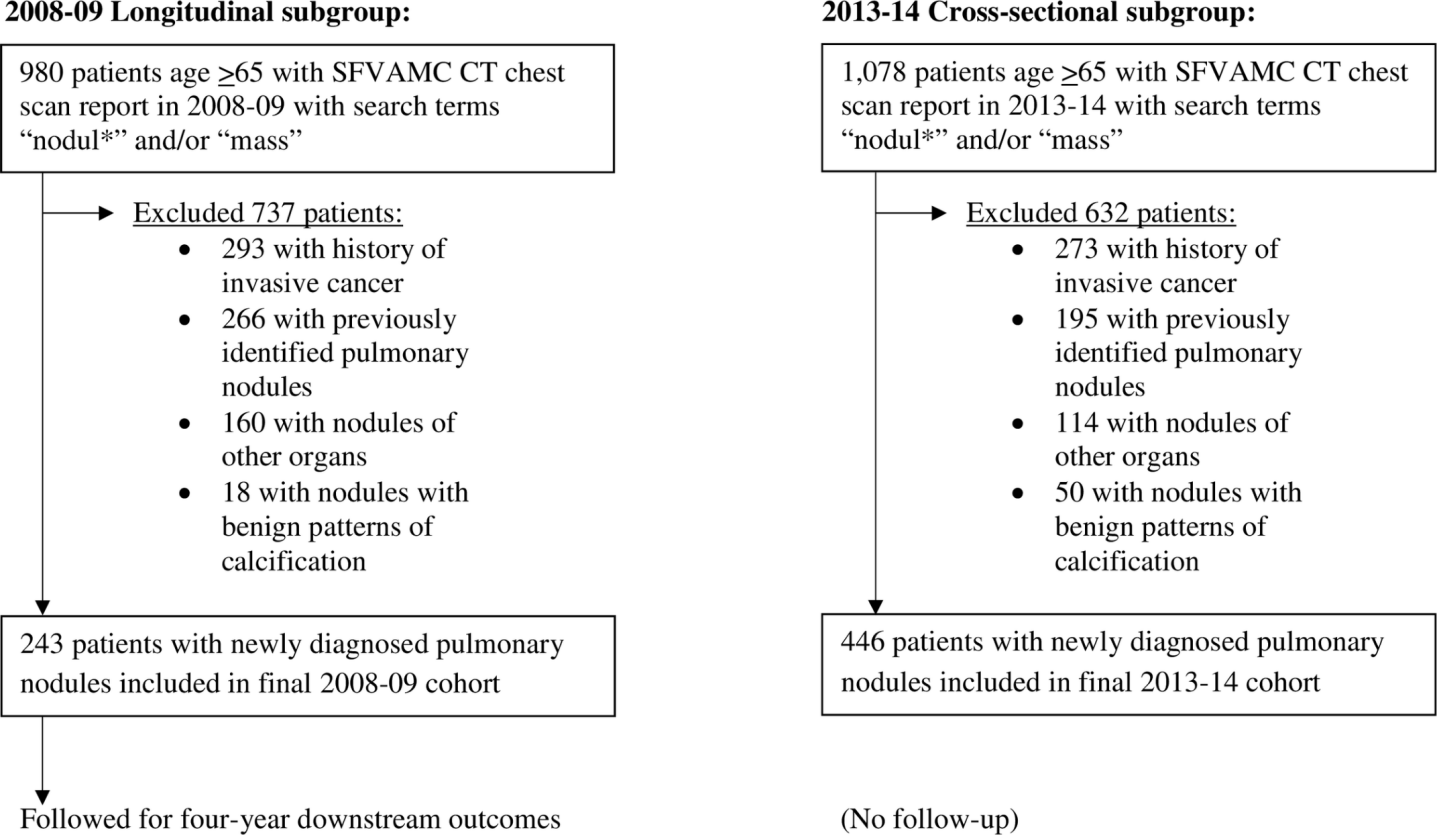
Exclusions used to define the final cohort of older veterans with a newly diagnosed pulmonary nodule at the SFVAMC in 2008–09 and 2013–14.

Our initial search identified 2,058 patients with CT chest reports that met the search terms. After manual review, 1,369 patients were excluded, resulting in a final cohort of 689 patients: 243 patients in 2008–09 and 446 patients in 2013–14 (Fig 1).

The University of California, San Francisco Institutional Review Board and the SFVAMC Research and Development Committee approved this study and provided a waiver of consent because obtaining informed consent from patients was not practical given the large number of required records for this minimal risk study. Identifiable data was abstracted from the medical record and deidentified prior to analysis.

### Data collection and measurement

#### Baseline characteristics

Age at the index CT scan was categorized as 65–74, 75–84, or ≥85 years. Comorbidity was assessed using the Charlson-Deyo Comorbidity Index (CCI) [8], a summary measure of 19 chronic diseases weighted according to their association with mortality [9]. Malignancy and metastatic disease did not contribute to the CCI in this study because patients with a history of invasive cancer were excluded. The CCI was calculated based on inpatient and outpatient VA claims during the 12 months prior to the index CT scan. Patients were categorized as having no comorbidity (CCI 0), average (CCI 1–3), or severe comorbidity (CCI ≥4).

Patients were categorized as having limited life expectancy (estimated <5 years) if age was ≥85 with CCI ≥1 or if age was ≥65 with CCI ≥4. Patients were categorized as having favorable life expectancy (estimated >10 years) if age was 65–74 with CCI 0. The remaining patients were categorized as having intermediate life expectancy. These categories have been used in previous studies to examine how extremes of health status influence cancer screening.[10–12] Smoking history at the index CT scan (never, former, current smoker) was abstracted from medical record notes.

Baseline pulmonary nodule characteristics were abstracted from the index radiology report and medical record. Patients were categorized as having an incidental nodule if the index CT scan was performed for a reason unrelated to pulmonary nodules (e.g., pulmonary embolism, aortic aneurysm evaluation), a symptom-detected nodule if the index scan was performed for symptoms potentially attributable to pulmonary nodules or suspected malignancy (e.g., cough, weight loss), or a screen-detected nodule if they underwent imaging for lung cancer screening or VA-related health screening (e.g., Agent Orange exposure). Nodule quality was categorized as solid, ground glass, or mixed. Nodule size for the largest dominant nodule was abstracted from the radiology report and categorized as ≤4 mm, >4–6 mm, >6–8 mm, and >8 mm according to Fleischner guideline size categories [3]. The presence of spiculation, dominant nodule in the upper lobe, multiple nodules, chest lymphadenopathy, and suspected infection was also determined.

#### Outcome variables

For the subgroup of patients with newly diagnosed pulmonary nodules in 2008–09, we linked VA and Medicare claims to capture nodule follow-up. Patients were followed for four years, or until the first diagnosis of invasive cancer or death.

We identified follow-up nodule imaging (CT chest and/or PET scans) and biopsy (transcutaneous, transbronchial, or surgical) using CPT and ICD-9 procedure codes. Medical records were reviewed to confirm that imaging and biopsies were follow-up of the index pulmonary nodule(s). Biopsy complications were also abstracted. Because some patients proceeded directly from index CT scan to biopsy without additional surveillance imaging, we examined receipt of any follow-up nodule imaging and/or biopsy as a composite primary outcome. Pulmonary nodule diagnostic outcomes were categorized as lung cancer, other type of cancer, or no cancer diagnosis.

### Statistical analysis

To evaluate differences across diagnosis year groups (2008–09 versus 2013–14), patient and nodule characteristics were compared using chi-square or Fisher’s exact tests.

For the patients with newly diagnosed pulmonary nodules in 2008–09, we performed univariable and multivariable Poisson regression models with robust error variances [13] to determine associations of patient and baseline nodule characteristics with receipt of nodule follow-up. The multivariable model adjusted for all patient and nodule characteristics except life expectancy since it is a composite predictor created from age and comorbidity. The multivariable model for life expectancy adjusted for all patient and nodule characteristics except age and comorbidity. For ordinal predictor variables, linear trend tests were also performed. In addition, we checked for an interaction effect between comorbidity/life expectancy and nodule size.

The threshold of *P* <0.05 was used to determine statistical significance for all two-sided comparisons. Analyses were conducted with SAS v9.2 (SAS Institute, Cary, NC).

## Results

### Patient and nodule characteristics

Our cohort included a total of 689 veterans age ≥65 with a newly diagnosed pulmonary nodule (243 veterans in 2008–09, 446 veterans in 2013–14). Median age was 73 (IQR 68–81) in 2008–09 and 70 (IQR 66–76) in 2013–14. In 2008–09, 129 patients (53.1%) had a CCI of 0 and 22 (9.0%) had a CCI ≥4. In 2013–14, 197 patients (44.2%) had a CCI of 0 and 44 (9.9%) had a CCI ≥4 (*P* = 0.08) (Table 1). The most common comorbidities were COPD, diabetes, and congestive heart failure. In 2008–09, 75 (30.9%) of patients had a favorable life expectancy while 33 (13.6%) had a limited life expectancy. In 2013–14, 142 patients (31.8%) had a favorable life expectancy while 67 (15.0%) had a limited life expectancy (*P* = 0.80). The majority of patients were former smokers (65.4% in 2008–09, 58.2% in 2013–14).

**Table 1:** Patient and baseline pulmonary nodule characteristics in veterans age ≥65 with newly diagnosed pulmonary nodule(s) by diagnosis year group (N = 689).

	2008–09	2013–14	^b^
	(n = 243)	(n = 446)	
Characteristic^a^	No. (%)	No. (%)	*P*
Age at pulmonary nodule diagnosis, year			0.001
65–74	132 (54.3)	306 (68.6)	
75–84	82 (33.7)	106 (23.8)	
≥85	29 (11.9)	34 (7.6)	
Charlson Comorbidity Index			0.08
0	129 (53.1)	197 (44.2)	
1–3	92 (37.9)	205 (46.0)	
≥4	22 (9.0)	44 (9.9)	
Selected Charlson comorbidities			
COPD	53 (21.8)	124 (27.8)	0.09
Diabetes (with/without complications)	40 (16.4)	85 (19.1)	0.40
Congestive heart failure	32 (13.2)	53 (11.9)	0.62
Moderate or severe renal disease	28 (11.5)	49 (11.0)	0.83
Cerebrovascular disease	21 (8.6)	30 (6.7)	0.36
Peripheral vascular disease	18 (7.4)	48 (10.8)	0.15
Myocardial infarct	14 (5.8)	21 (4.7)	0.55
Mild liver disease	4 (1.7)	7 (1.6)	1.00^c^
Ulcer disease	2 (0.8)	10 (2.2)	0.23^c^
Moderate or severe liver disease	2 (0.8)	2 (0.5)	0.62^c^
Connective tissue disease	1 (0.4)	6 (1.4)	0.43^c^
Dementia	1 (0.4)	5 (1.1)	0.67^c^
Hemiplegia	1 (0.4)	0 (0)	0.35^c^
AIDS	0 (0)	5 (1.1)	0.17^c^
Life expectancy			0.80
Favorable	75 (30.9)	142 (31.8)	
Intermediate	135 (55.5)	237 (53.1)	
Limited	33 (13.6)	67 (15.0)	
Male gender	237 (97.5)	432 (96.9)	0.62
Race			0.01
White	183 (76.9)	364 (84.9)	
Black	27 (11.3)	40 (9.3)	
Other	28 (11.8)	25 5.8)	
Married	98 (41.0)	171 (39.1)	0.63
Lived in ZCTA in which ≥25% of adults had a college education	169 (72.5)	281 (64.8)	0.04
Median annual income of ZCTA			0.19
Lowest tertile	68 (29.2)	155 (35.7)	
Middle tertile	80 (34.3)	144 (33.2)	
Highest tertile	85 (36.5)	135 (31.1)	
Smoking history			0.17
Never smoker	41 (16.9)	88 (19.8)	
Former smoker	159 (65.4)	259 (58.2)	
Current smoker	43 (17.7)	98 (22.0)	
Reason for nodule detection			<0.001
Incidental	104 (42.8)	221 (49.6)	
Vascular evaluation^d^	36 (14.8)	59 (13.2)	
Perioperative	32 (13.2)	39 (8.7)	
Abdominal CT	13 (5.3)	48 (10.8)	
Cardiac evaluation^e^	3 (1.2)	24 (5.4)	
TAVR	0 (0)	24 (5.4)	
Other	20 (8.2)	27 (6.1)	
Symptom-detected	136 (56.0)	157 (35.2)	
Screen-detected	3 (1.2)	68 (15.3)	
Lung cancer screening	2 (0.1)	42 (9.4)	
VA-related screening	1 (0.04)	26 (5.8)	
Nodule quality			0.09
Solid	207 (85.2)	388 (87.0)	
Ground glass	16 (6.6)	14 (3.1)	
Mixed	20 (8.2)	44 (9.9)	
Maximum nodule size			0.03
≤4 mm	110 (45.3)	229 (51.4)	
4–6 mm	54 (22.2)	95 (21.3)	
>6–8 mm	16 (6.6)	45 (10.1)	
>8 mm	63 (25.9)	77 (17.3)	
Spiculated dominant nodule	18 (7.4)	28 (6.3)	0.57
Upper lobe location	101 (41.6)	190 (42.6)	0.79
Multiple nodules	137 (56.4)	307 (68.8)	0.001
Suspicious chest lymphadenopathy	39 (16.1)	51 (11.4)	0.09
Infection suspected	26 (10.7)	59 (13.2)	0.34

Abbreviations: COPD, chronic obstructive pulmonary disorder; TAVR, transcatheter aortic valve replacement; ZCTA, zip code tabulation area.

^a^The following variables had missing data: race (3.2%), marital status (1.9%), ZCTA college education (3.2%), median annual income (3.2%), and smoking history (0.2%).

^b^Differences between 2008–09 and 2013–14 diagnosis year groups were tested using chi-square test, unless otherwise indicated.

^c^Differences between 2008–09 and 2013–14 diagnosis year groups tested using Fisher’s exact test.

^d^Vascular evaluation such as imaging for pulmonary embolism or aortic dissection.

^e^Cardiac evaluation such as imaging for coronary calcium.

Symptoms were the most common reason for nodule detection (56.0%) in 2008–09 and incidental findings (49.6%) were most common in 2013–14 (*P* <0.001). In 2008–09, incidental nodules were most commonly detected on CT chest scans to evaluate vasculature (14.8%, e.g., pulmonary embolism, aortic dissection) and perioperatively (13.2%). In 2013–14, incidental nodules were also identified on CT chest scans for new indications including transcatheter aortic valve replacement (TAVR) planning (5.4%) and other cardiac evaluation (5.4%). Screen-detected nodules increased from 1.2% in 2008–09 to 15.3% in 2013–14, primarily with the implementation of lung cancer screening. Of note, among patients with screen-detected nodules, only 2 patients (3%) in 2013–14 met criteria for limited life expectancy. From 2008–09 to 2013–14, pulmonary nodules were smaller in size with 67.5% of patients with nodules ≤6 mm in 2008–09 and 72.7% in 2013–14 (*P* = 0.03). Additionally, patients in 2013–14 were more likely to have multiple nodules (68.8%) compared to patients in 2008–09 (56.4%; *P* = 0.001).

### Nodule follow-up

**Fig 2:**
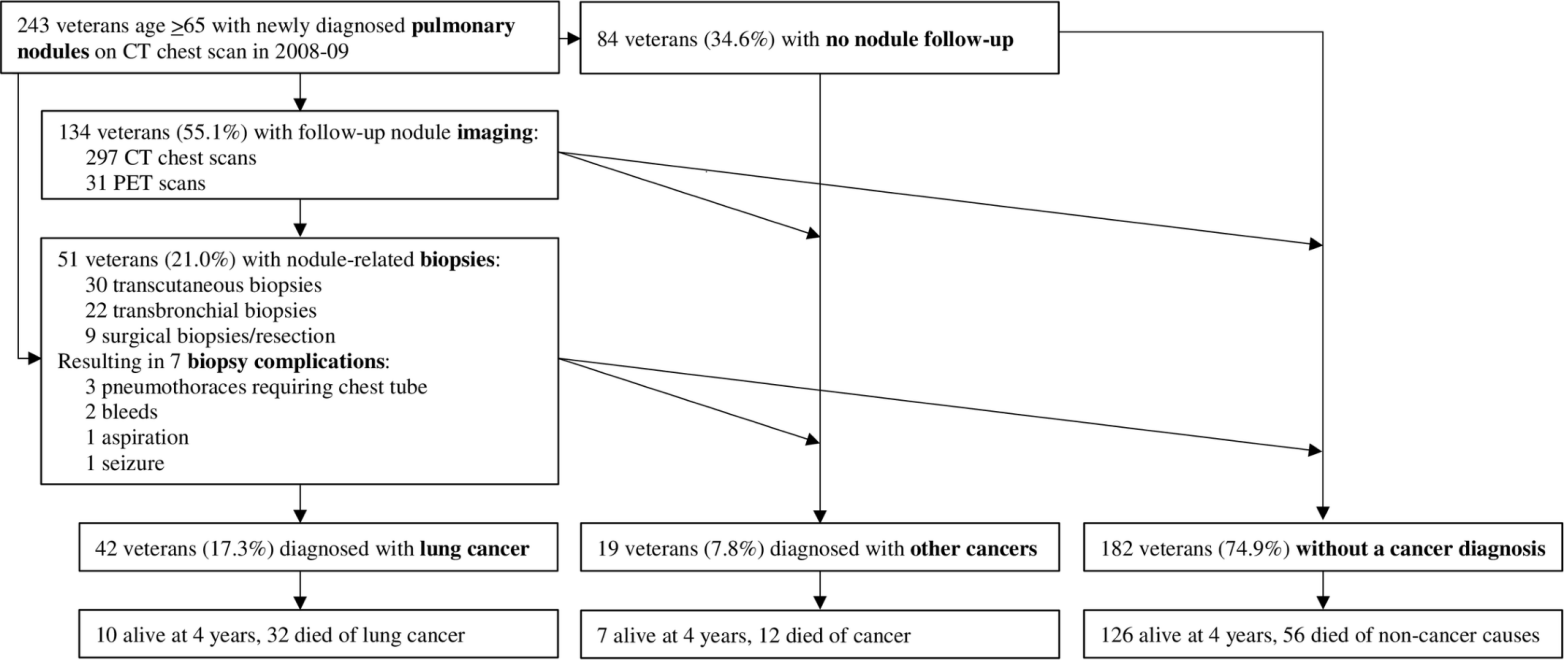
Four-year follow-up after pulmonary nodule diagnosis in veterans age ≥65 with newly diagnosed pulmonary nodules at the SFVAMC in 2008–09.

In the subgroup of 243 patients with newly diagnosed nodules in 2008–09, 134 patients (55.1%) underwent a total of 297 CT chest and 31 PET scans during four-year follow-up (Fig 2). A total of 51 patients (21.0%) underwent a nodule-related biopsy during four-year follow-up, the majority of which were transcutaneous. Median time from index CT scan to first follow-up nodule imaging or biopsy was 11.5 months (IQR 3.0 months to not reached). The biopsy complication rate was 13.7%: 3 pneumothoraces requiring chest tube, 2 bleeds, 1 aspiration, and 1 post-procedure seizure. Ultimately, 42 patients overall were diagnosed with lung cancer (12.5% of patients with incidental nodules, 21.3% of patients symptom-detected nodules) and 19 were diagnosed with other types of cancer (6.7% of incidental nodules, 8.1% of symptom-detected nodules), leaving 182 patients (74.9%) without cancer. The most common cause of death was non-cancer related (23.0%), while 13.2% died of lung cancer and 4.9% died of other cancers.

In unadjusted analyses (Table 2), there was no association between age and the receipt of follow-up nodule imaging and/or biopsy. Nodule follow-up was less likely with increasing comorbidity (CCI 1–3: RR 0.79, 95% CI 0.63–0.98; CCI ≥4: RR 0.59, 95% CI 0.35–0.99; test for linear trend *P* = 0.05). Patients with limited life expectancy were also less likely to undergo nodule follow-up compared to those with favorable life expectancy (RR 0.64, 95% CI 0.43–0.96). In addition, nodule follow-up was more likely for former (RR 1.74, 95% CI 1.14–2.64) and current smokers (RR 2.03, 95% CI 1.31–3.16) compared to never smokers (test for linear trend *P* = 0.002) as well as for patients with high-risk nodule characteristics (i.e., larger size and spiculation). In contrast, nodule follow-up was less likely for patients with multiple nodules and suspected infection. There were no differences in nodule follow-up according to other demographic variables.

**Table 2:** Patient and baseline nodule characteristics associated with receipt of any nodule imaging and/or biopsy during four-year follow-up in veterans age ≥65 with newly diagnosed pulmonary nodule(s) in 2008–09 (n = 243).

	Receipt of any follow-up nodule imaging and/or biopsy^a^		
	No. (%)	Unadjusted RR (95% CI)	Adjusted RR^b^ (95% CI)
Age			
65–74	83 (62.9)	1.00	1.00
75–84	48 (58.5)	0.93 (0.74–1.17)	0.96 (0.75–1.22)
≥85	17 (58.6)	0.93 (0.67–1.30)	1.01 (0.68–1.49)
CCI			
0	89 (69.0)	1.00^d^	1.00
1–3	50 (54.4)	0.79 (0.63–0.98)	0.85 (0.68–1.08)
≥4	9 (40.9)	0.59 (0.35–0.99)	0.61 (0.39–0.95)
Life expectancy^c^			
Favorable	53 (70.7)	1.00^d^	1.00
Intermediate	80 (59.3)	0.84 (0.68–1.03)	0.85 (0.69–1.04)
Limited	15 (45.5)	0.64 (0.43–0.96)	0.69 (0.48–1.01)
Male gender	144 (60.8)	0.91 (0.51–1.62)	1.02 (0.50–2.10)
Race			
White	111 (60.7)	1.00	1.00
Black	19 (70.4)	1.16 (0.88–1.52)	1.16 (0.87–1.54)
Other	16 (57.1)	0.94 (0.67–1.33)	1.06 (0.79–1.43)
Married	56 (57.1)	0.92 (0.74–1.13)	0.96 (0.77–1.19)
Lived in ZCTA in which ≥25% of adults had a college education	103 (61.0)	1.05 (0.83–1.34)	1.12 (0.84–1.51)
Median annual income of ZCTA			
Lowest tertile	41 (60.3)	1.00	1.00
Middle tertile	47 (58.8)	0.97 (0.75–1.27)	0.84 (0.61–1.14)
Highest tertile	52 (61.2)	1.01 (0.78–1.31)	0.92 (0.66–1.28)
Smoking history			
Never smoker	15 (36.6)	1.00^d^	1.00^d^
Former smoker	101 (63.5)	1.74 (1.14–2.64)	1.60 (1.05–2.42)
Current smoker	32 (74.4)	2.03 (1.31–3.16)	1.67 (1.08–2.60)
Symptom-detected nodule	84 (61.8)	1.03 (0.84–1.27)	0.92 (0.73–1.15)
Nodule quality			
Solid	127 (61.4)	1.00	1.00
Ground glass	9 (56.3)	0.92 (0.59–1.43)	1.16 (0.75–1.78)
Mixed	12 (60.0)	0.98 (0.67–1.42)	1.43 (1.00–2.05)
Maximum nodule size			
≤4 mm	52 (47.3)	1.00^d^	1.00^d^
>4–6 mm	31 (57.4)	1.21 (0.90–1.64)	1.21 (0.89–1.63)
>6–8 mm	12 (75.0)	1.59 (1.12–2.24)	1.47 (0.98–2.19)
>8 mm	53 (84.1)	1.78 (1.42–2.23)	1.54 (1.16–2.05)
Spiculation	17 (94.4)	1.62 (1.39–1.90)	1.29 (1.01–1.63)
Upper lobe location	67 (66.3)	1.16 (0.95–1.42)	1.13 (0.91–1.39)
Multiple nodules	74 (54.0)	0.77 (0.63–0.94)	0.93 (0.75–1.14)
Chest lymphadenopathy	28 (71.8)	1.22 (0.97–1.53)	1.17 (0.89–1.54)
Suspected infection	7 (26.9)	0.41 (0.22–0.79)	0.55 (0.29–1.07)

Abbreviations: CCI, Charlson Comorbidity Index; RR, relative risk; ZCTA, zip code tabulation area.

^a^Using Poisson regression with robust error variances.

^b^Adjusted models included all patient and nodule characteristics except life expectancy.

^c^Adjusted model for life expectancy included all patient and nodule characteristics except age and CCI.

^d^Test for linear trend *P* < 0.05.

In adjusted analyses (Table 2), the association between comorbidity and receipt of nodule follow-up remained statistically significant only for CCI ≥4 compared to CCI 0 (adjusted RR 0.61, 95% CI 0.39–0.95). The association with CCI 1–3 no longer reached statistical significance (adjusted RR 0.85, 95% CI 0.68–1.08). Similarly, the association with limited life expectancy no longer reached statistical significance (adjusted RR 0.69, 95% CI 0.48–1.01) after adjustment. Smoking history, nodule size >8 mm, and spiculation remained strong predictors of nodule follow-up in the adjusted analyses. In the adjusted analysis, patients with mixed nodules were more likely to receive nodule follow-up than patients with solid nodules (adjusted RR 1.43, 95% CI 1.00–2.05, *P* = 0.05).

**Fig 3:**
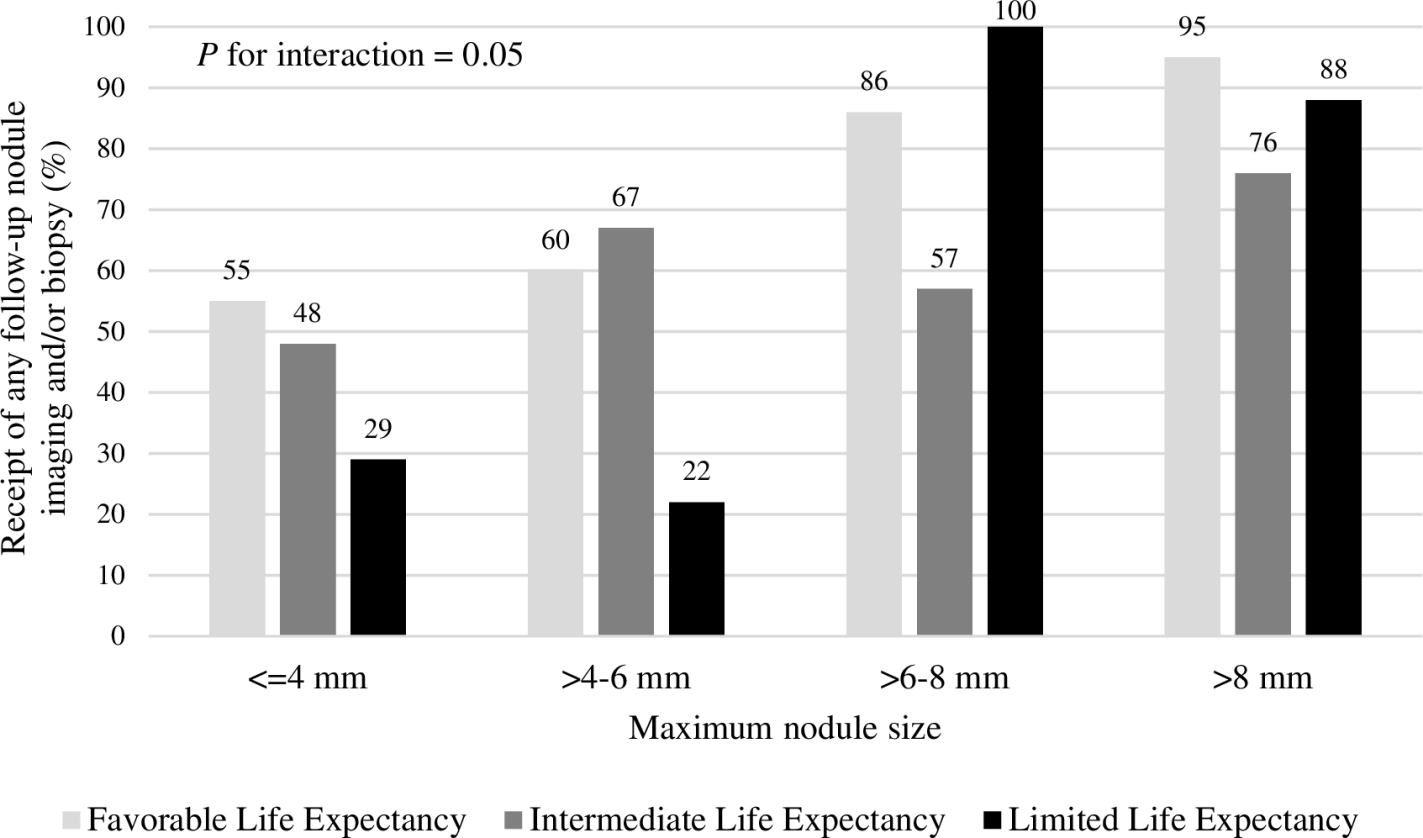
Receipt of any follow-up nodule imaging and/or biopsy during four-year follow-up in older veterans with newly diagnosed pulmonary nodules by life expectancy and nodule size. Patients were categorized as having limited life expectancy if age was ≥85 with CCI ≥1 or if age was ≥65 with CCI ≥4. Patients were categorized as having favorable life expectancy if age was 65–74 with CCI 0. The remaining patients were categorized as having intermediate life expectancy.

In addition, an interaction effect of life expectancy and nodule size on nodule follow-up was detected (*P* = 0.05) such that patients with limited life expectancy were less likely to receive follow-up only if they had smaller nodules ≤6 mm (Fig 3). In contrast, for patients with nodules >6 mm, there was no difference in nodule follow-up by life expectancy.

## Discussion

We found that the number of older veterans diagnosed with new pulmonary nodules almost doubled from 2008–09 to 2013–14, which reflects both the implementation of lung cancer screening [2] and increased imaging use for new indications such as TAVR planning [14] and cardiac evaluation [15]. Nodules diagnosed in 2013–14 were smaller as well. This increase in patients with nodules was true even among those with severe comorbidity and limited life expectancy. Overall, patients with severe comorbidity and limited life expectancy were less likely to receive nodule follow-up. However, there was an interaction effect of nodule size with comorbidity and life expectancy; severe comorbidity and limited life expectancy decreased follow-up in patients with smaller nodules (≤6 mm) but not in those with larger nodules (>6 mm).

To our knowledge, this is the first study to examine the impact of comorbidity and life expectancy on pulmonary nodule follow-up in an older population. Given the immediate and potentially substantial harms of intensive pulmonary nodule surveillance and invasive procedures, decreased nodule follow-up among patients with severe comorbidity and limited life expectancy may represent appropriate individualization of nodule evaluation where the risks of nodule follow-up outweigh the chance of benefit. Of note, the majority of patients with screen-detected nodules had favorable or intermediate life expectancy with only 3% having limited life expectancy. This is consistent with the USPSTF recommendation to discontinue lung cancer screening among patients with health problems that substantially limit their life expectancy [2].

While it is not known how life expectancy impacts the likelihood of harms from pulmonary nodule follow-up, the potential harms of nodule follow-up in the general population are well documented and a few studies have described harms according to age and comorbidity. In a study of transcutaneous biopsy, the risk of pneumothorax requiring chest tube was estimated at 6.6% with an overall 15% risk of any pneumothorax [16]. In studies of transbronchial biopsy, the overall complication rate ranged from 1.4–7.1% [17, 18]. Studies on the association between older age and transbronchial biopsy complications have been mixed. While older age was not associated with increased risk of transbronchial biopsy complications in a UK study [18], older age was associated with escalation of level of care after the procedure (i.e., hospital admission, ICU transfer) in a national US study [17] and with biopsy-related hemorrhage in a study of community hospitals in Florida [19]. Additionally, patients with renal failure and cirrhosis were at increased risk of hemorrhage while those with COPD were at increased risk of pneumothorax from transbronchial biopsy [19].

Potential harms of nodule follow-up are not limited to procedural complications. In a multicenter study of patients with incidental nodules, 26% of patients reported clinically significant nodule-related distress [20], which highlights the important psychosocial impact of nodule follow-up [21]. Misperceptions about nodules, the natural history, and likelihood of cancer were also common [20]. For example, only 25% of patients accurately estimated their risk of lung cancer and 71% were unaware of the possibility of indolent tumors. In that study, older patients were not more likely to report nodule-related distress [20], but the impact of comorbidity and life expectancy on nodule-related distress remains unknown. Overdiagnosis of indolent tumors [22], which may otherwise not cause clinical symptoms, is an additional potential harm of nodule follow-up. The potential harm of overdiagnosis is particularly important to consider for patients with limited life expectancy, where competing causes of death from other comorbidities may limit the potential benefits of nodule evaluation and possible subsequent cancer treatment [23, 24].

The potential harms of nodule follow-up are especially salient when the nodules detected are smaller with a lower risk of lung cancer [3, 4, 6], which may tip the balance from benefits towards possible harm. Indeed, we found that the impact of comorbidity and life expectancy on nodule follow-up differed by nodule size such that comorbidity and limited life expectancy were associated with decreased nodule follow-up only for patients with smaller nodules. We hypothesize that providers in our study were more comfortable deferring nodule follow-up for these smaller nodules given their lower risk of lung cancer [3, 4, 6]. However, for larger nodules, patients received nodule follow-up regardless of life expectancy. While this may be appropriate given the increased risk of lung cancer with increasing nodule size [3, 4], further research is needed to determine how incorporating comorbidity and life expectancy into decision-making for nodules of all sizes may impact patient outcomes.

Older age alone was not associated with decreased nodule follow-up in our study of veterans age ≥65. This finding differs from a retrospective study of veterans of all ages (mean age 66, SD 11 years) diagnosed with pulmonary nodules in 2003–06 [6]. In that study, older age was associated with less intensive nodule follow-up than recommended by guidelines [3]. However, without comprehensively adjusting for comorbidities, which are more common with older age, their result is difficult to interpret since residual confounding may be present. Since the risk of lung cancer increases with age [25], our study’s lack of a potential age bias in nodule follow-up in both the unadjusted and adjusted analyses is reassuring.

In contrast to age, smoking history and nodule characteristics (e.g., size, spiculation) were strong predictors of nodule follow-up. These findings are consistent with the 2005 Fleischner Society guidelines [3] for management of incidental pulmonary nodules where recommendations are primarily based on nodule size and smoking history. While the 2005 Fleischner guidelines state that the “patient’s age and the presence of comorbid conditions should influence management recommendations,” [3] no specific guidance on how to do so is provided. Fortunately, the 2017 revision of the Fleischner guidelines [4] allows for more patient and provider discretion in nodule management by providing a range of follow-up intervals rather than precise times. In addition, routine follow-up for nodules <6 mm, which may represent overdiagnosis, is no longer recommended unless high risk features are present. Together, these improvements to the guidelines may help decrease the potential harms of nodule follow-up among patients with severe comorbidity and limited life expectancy while preserving the potential clinical benefit for older adults in good health with favorable life expectancy who may benefit most. We found that patients with mixed nodules were more likely to receive nodule follow-up than patients with solid nodules. This is surprising since the risk of invasive lung cancer is generally lower with mixed nodules depending on the size of the solid component compared to solid nodules [26]. The association between mixed nodules and increased nodule follow-up only emerged after adjustment for patient and nodule characteristics, suggesting the presence of negative confounding that attenuated the true association.

Our study has a number of strengths, including a comparison of patient and nodule characteristics over time and a comprehensive examination of the impact of age, comorbidity, and life expectancy on pulmonary nodule evaluation.

However, it also has several limitations. First, as a single center study of pulmonary nodule evaluation at an urban VA medical center, practice patterns may differ from other VA and non-VA medical centers. However, the high rates of older adults, current or former smokers, and severe comorbidity among veterans make them an ideal population for our research questions. Second, due to the small number of biopsy complications, we did not have sufficient power to examine predictors of biopsy complications in this study. Lastly, we examined four-year follow-up only for the subgroup of patients with newly diagnosed pulmonary nodules in 2008–09 with outcomes through 2013. Data are not yet available to examine four-year follow-up for the subgroup with newly diagnosed pulmonary nodules in 2013–14 after implementation of lung cancer screening and the Lung-RADS guideline. However, since over 90% of the new pulmonary nodules identified in our 2013–14 subgroup were unrelated to lung cancer screening, future studies of nodule management in routine clinical care should examine incidental and symptom-detected nodules in addition to screen-detected nodules.

## Conclusions

As the number of older patients diagnosed with pulmonary nodules increases over time, nodule follow-up can be individualized by considering comorbidity and life expectancy for all patients, not just those with smaller nodules. Pulmonary nodule guidelines, for both incidental nodules and screen-detected nodules, need to be more explicit about how to best incorporate comorbidity and life expectancy information together with smoking history and nodule characteristics to determine when and how to follow-up a pulmonary nodule. Better individualized care for older adults with pulmonary nodules will maximize benefits and reduce harms of this increasingly common clinical management challenge.

## Supporting information

S1 File Manuscript dataset. This spreadsheet includes the data for the variables examined in this manuscript.
(XLSX)

S2 File Dataset contents. This file provides supplemental information about the variables included in the manuscript dataset.
(DOCX)
