# Supplementary material for: Age, comorbidity, life expectancy, and pulmonary nodule follow-up in older veterans
Source: PLoS One. 2018 Jul 25;13(7):e0200496. doi: 10.1371/journal.pone.0200496 (PMC6059441; doi:10.1371/journal.pone.0200496)
Supplement: S2 File — This file provides supplemental information about the variables included in the manuscript dataset. (DOCX) [file pone.0200496.s002.docx]

The CONTENTS Procedure

Data Set Name NODULE_PLOSONE Observations 689

Member Type DATA Variables 48

Engine V9 Indexes 0

Created 06/04/2018 09:06:32 Observation Length 440

Last Modified 06/04/2018 09:06:32 Deleted Observations 0

Protection Compressed NO

Data Set Type Sorted NO

Label

Data Representation WINDOWS_64

Encoding wlatin1 Western (Windows)

The CONTENTS Procedure

Variables in Creation Order

| # | Variable | Type | Len | Format | Informat | Label |
| --- | --- | --- | --- | --- | --- | --- |
|  |  |  |  |  |  |  |
| 1 | cohort | Char | 7 | $7.00 | $7.00 | cohort |
| 2 | age | Char | 5 | $5.00 | $5.00 | age |
| 3 | charlson | Char | 3 | $3.00 | $3.00 | charlson |
| 4 | copd | Num | 8 | BEST. |  | copd |
| 5 | diabetes | Num | 8 | BEST. |  | diabetes |
| 6 | chf | Num | 8 | BEST. |  | chf |
| 7 | renal | Num | 8 | BEST. |  | renal |
| 8 | cvd | Num | 8 | BEST. |  | cvd |
| 9 | pvd | Num | 8 | BEST. |  | pvd |
| 10 | mi | Num | 8 | BEST. |  | mi |
| 11 | mildliver | Num | 8 | BEST. |  | mildliver |
| 12 | ulcer | Num | 8 | BEST. |  | ulcer |
| 13 | modsevliver | Num | 8 | BEST. |  | modsevliver |
| 14 | contissuedz | Num | 8 | BEST. |  | contissuedz |
| 15 | dementia | Num | 8 | BEST. |  | dementia |
| 16 | hemiplegia | Num | 8 | BEST. |  | hemiplegia |
| 17 | aids | Num | 8 | BEST. |  | aids |
| 18 | lifeexpect | Char | 12 | $12.00 | $12.00 | lifeexpect |
| 19 | male | Num | 8 | BEST. |  | male |
| 20 | racegrp | Char | 5 | $5.00 | $5.00 | racegrp |
| 21 | married | Num | 8 | BEST. |  | married |
| 22 | zctacollege | Num | 8 | BEST. |  | zctacollege |
| 23 | zctaincome | Char | 14 | $14.00 | $14.00 | zctaincome |
| 24 | smoking | Char | 14 | $14.00 | $14.00 | smoking |
| 25 | nodtype | Char | 16 | $16.00 | $16.00 | nodtype |
| 26 | incidentalcat | Char | 19 | $19.00 | $19.00 | incidentalcat |
| 27 | screencat | Char | 20 | $20.00 | $20.00 | screencat |
| 28 | nodquality | Char | 11 | $11.00 | $11.00 | nodquality |
| 29 | nodsize | Char | 7 | $7.00 | $7.00 | nodsize |
| 30 | spiculated | Num | 8 | BEST. |  | spiculated |
| 31 | upperlobe | Char | 1 | $1.00 | $1.00 | upperlobe |
| 32 | multinod | Char | 1 | $1.00 | $1.00 | multinod |
| 33 | chestlad | Num | 8 | BEST. |  | chestlad |
| 34 | infxn | Num | 8 | BEST. |  | infxn |
| 35 | sxdetected | Num | 8 | BEST. |  | sxdetected |
| 36 | anyfuimaging | Num | 8 | BEST. |  | anyfuimaging |
| 37 | anybx | Num | 8 | BEST. |  | anybx |
| 38 | anyfu | Num | 8 | BEST. |  | anyfu |
| 39 | ctscan | Num | 8 | BEST. |  | ctscan |
| 40 | petscan | Num | 8 | BEST. |  | petscan |
| 41 | transcutbx | Num | 8 | BEST. |  | transcutbx |
| 42 | transbroncbx | Num | 8 | BEST. |  | transbroncbx |
| 43 | otherbx | Num | 8 | BEST. |  | otherbx |
| 44 | bxcomplication | Char | 19 | $19.00 | $19.00 | bxcomplication |
| 45 | lungcancer | Num | 8 | BEST. |  | lungcancer |
| 46 | otherca | Num | 8 | BEST. |  | otherca |
| 47 | death | Num | 8 | BEST. |  | death |
| 48 | causeofdeath | Char | 23 | $23.00 | $23.00 | causeofdeath |
